# Role of aldo-keto reductases and other doxorubicin pharmacokinetic genes in doxorubicin resistance, DNA binding, and subcellular localization

**DOI:** 10.1186/1471-2407-12-381

**Published:** 2012-08-31

**Authors:** Allan D Heibein, Baoqing Guo, Jason A Sprowl, David A MacLean, Amadeo M Parissenti

**Affiliations:** 1 Graduate Program in Biology, Laurentian University, Sudbury, ON, Canada; 2 Regional Cancer Program, Sudbury Regional Hospital, Sudbury, ON, P3E 5J1, Canada; 3 Graduate Program in Biomolecular Science, Laurentian University, Sudbury, ON, Canada; 4 Division of Medical Sciences, Northern Ontario School of Medicine, Sudbury, ON, Canada; 5 Divison Of Oncology, Faculty of Medicine, University of Ottawa, Ottawa, Canada

**Keywords:** Doxorubicin, Resistance, Gene profiling, PharmGKB, Pharmacokinetics, Aldo-keto reductases, Cytotoxicity, Lysosome, DNA-binding, Drug localization

## Abstract

**Background:**

Since proteins involved in chemotherapy drug pharmacokinetics and pharmacodynamics have a strong impact on the uptake, metabolism, and efflux of such drugs, they likely play critical roles in resistance to chemotherapy drugs in cancer patients.

**Methods:**

To investigate this hypothesis, we conducted a whole genome microarray study to identify difference in the expression of genes between isogenic doxorubicin-sensitive and doxorubicin-resistant MCF-7 breast tumour cells. We then assessed the degree of over-representation of doxorubicin pharmacokinetic and pharmacodynamic genes in the dataset of doxorubicin resistance genes.

**Results:**

Of 27,958 Entrez genes on the array, 7.4 per cent or 2,063 genes were differentially expressed by ≥ 2-fold between wildtype and doxorubicin-resistant cells. The false discovery rate was set at 0.01 and the minimum p value for significance for any gene within the “hit list” was 0.01. Seventeen and 43 per cent of doxorubicin pharmacokinetic genes were over-represented in the hit list, depending upon whether the gene name was identical or within the same gene family, respectively. The most over-represented genes were within the 1C and 1B families of aldo-keto reductases (AKRs), which convert doxorubicin to doxorubicinol. Other genes convert doxorubicin to other metabolites or affect the influx, efflux, or cytotoxicity of the drug. In further support of the role of AKRs in doxorubicin resistance, we observed that, in comparison to doxorubicin, doxorubincol exhibited dramatically reduced cytotoxicity, reduced DNA-binding activity, and strong localization to extra nuclear lysosomes. Pharmacologic inhibition of the above AKRs in doxorubicin-resistant cells increased cellular doxorubicin levels, restored doxorubicin cytotoxicity and re-established doxorubicin localization to the nucleus. The properties of doxorubicinol were unaffected.

**Conclusions:**

These findings demonstrate the utility of using curated pharmacokinetic and pharmacodynamic knowledge bases to identify highly relevant genes associated with doxorubicin resistance. The induction of one or more of these genes was found to be correlated with changes in the drug’s properties, while inhibiting one specific class of these genes (the AKRs) increased cellular doxorubicin content and restored drug DNA binding, cytotoxicity, and subcellular localization.

## Background

Doxorubicin is a DNA-binding, topoisomerase II inhibitor [1,2], which is among the most effective chemotherapy drugs in cancer treatment [1,3]. However, intrinsic or acquired resistance to doxorubicin in patient tumours is common, resulting in treatment failure and disease progression. Multiple mechanisms for doxorubicin resistance have been identified *in vitro*, such as the increased expression of drug transporters [4-7], alterations in doxorubicin metabolism [8] or localization [9,10], and defects in the drug’s ability to induce apoptosis [11]. Unfortunately, progress in restoring drug sensitivity for drug-resistant tumours, particularly by inhibiting drug efflux transporters, has been incremental at best [12,13]. This limited progress demands that a more nuanced approach be taken, including the identification of all proteins that likely affect the pharmacokinetics and pharmacodynamics of doxorubicin.

Genome profiling is a method that can provide data on gene expression and/or allelic variations across biological samples, often using whole genome approaches. This promises to be a great aid to oncologists in identifying and treating drug-resistant tumours. Unfortunately, this task is a difficult one, given the variability associated with patient data sets and the large number of “false positives” inherent in such approaches from by-stander effects. One method to improve the identification of genes relevant to a specific phenomenon such as doxorubicin resistance is to pair knowledge of metabolic or signal transduction pathways to gene expression data [14]. In this study, we use full genome microarray analysis to compare gene expression between MCF-7 cells selected for maximal resistance to doxorubicin (MCF-7_DOX2-12_ cells) and equivalent cells selected for the same number of passages in the absence of drug (MCF-7_CC12_ cells). After identifying genes having altered expression in doxorubicin-resistant cells, we then used a well-known, curated pharmacogenomics knowledgebase (PharmGKB) to identify which of these genes play a role in doxorubicin pharmacokinetics or pharmacodynamics, as these were more likely to have a direct effect on doxorubicin efficacy. This combination of full genome microarray analysis identifying genes differentially expressed upon acquisition of doxorubicin resistance with an assessment of over-representation of doxorubicin pharmacokinetic or pharmacokinetic genes in the dataset provided significant insight into new pathways associated with doxorubicin resistance. Moreover, extensive comparisons between the biochemical properties of doxorubicin and one of its metabolites (doxorubicinol) provided us with significant insight into how a simple hydroxylation reaction can strongly affect the biochemical and cellular properties of doxorubicin, including dramatically reduced cytotoxicity, diminished DNA- binding activity, altered cellular accumulation of the drug and altered subcellular localization.

## Results

### Differentially expressed genes upon acquisition of doxorubicin resistance

Using full genome Agilent microarrays and Partek Genomics Suite, 2063 genes from a total of 27958 Entrez genes on the array (7.4%) were found to be differentially expressed by ≥2-fold between MCF-7_CC12_ cells MCF-7_DOX2-12_ cells. The false discovery rate was set at 0.01 and the minimum p value for significance for any gene within the “hit list” was 0.01. The microarray data was deposited in the NCBI Gene Expression Omnibus (GEO) database, accession number GSE27254) in accordance with MIAME standards [15]. Access to the microarray data can be obtained via the following url: http://www.ncbi.nlm.nih.gov/geo/query/acc.cgi?token=dbezngycywquuhm&acc=GSE27254.

**Figure 1:**
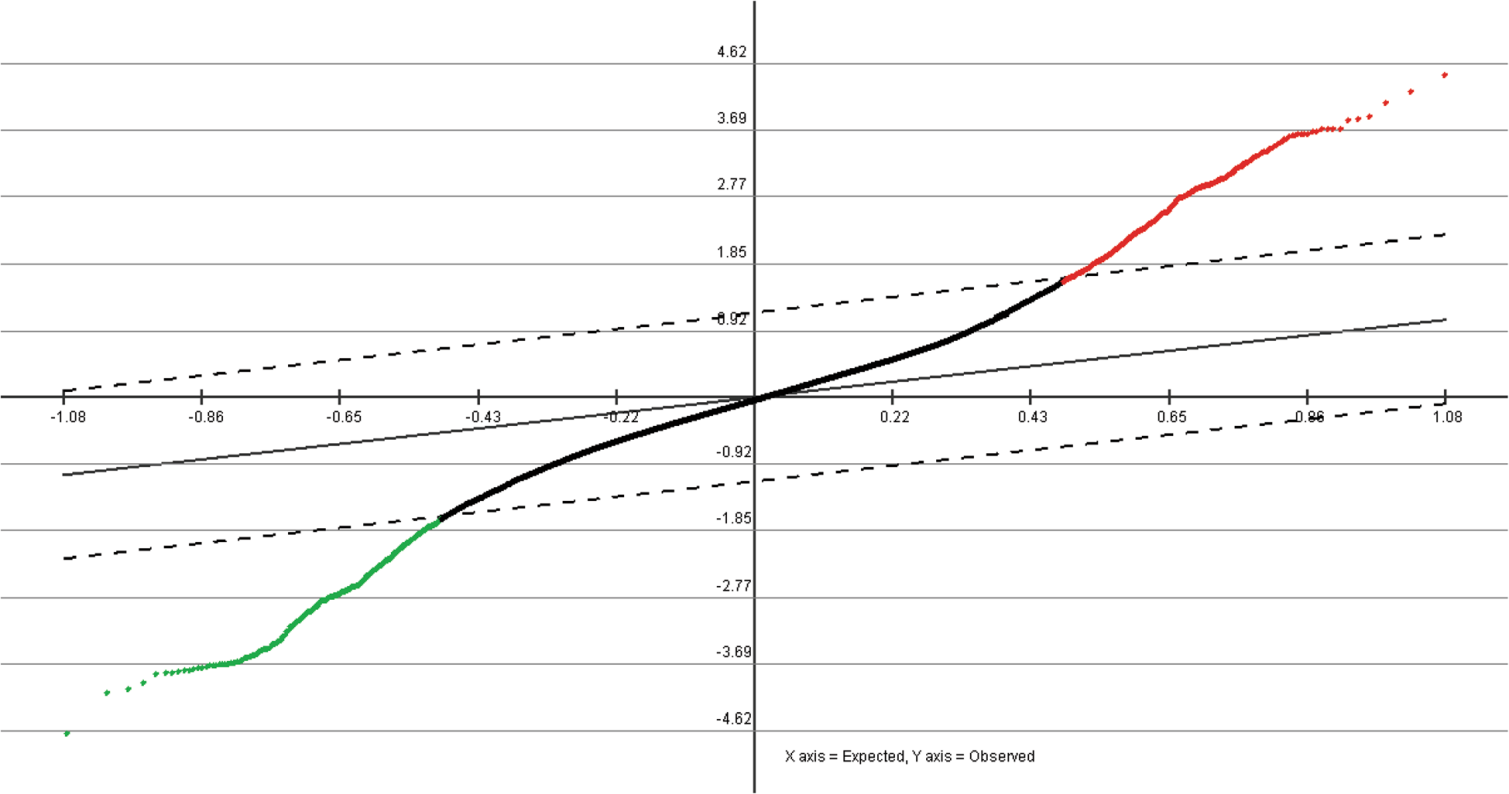
**Significance Analysis of Microarrays (SAM) graph for MCF-7**_**DOX2-12**_**and MCF-7**_**CC112**_**cells.** Red or green points denote genes with significant over- or under-expression in MCF-7_DOX2-12_ cells compared to MCF-7_CC12_ cells, while black points represent genes that do not show altered expression.

The identification of thousands of genes changing expression upon selection of MCF-7 cells for doxorubicin resistance was similar to the numbers of genes observed when these cells were selected for resistance to other chemotherapy agents (data not shown). These findings indicate that a significant amount of the transcriptome appears altered as these cells are selected for doxorubicin resistance. In addition to providing candidate genes that may be involved in doxorubicin resistance, the microarray data served to demonstrate that MCF-7_DOX2_ cells at selection dose 12 and MF-7_CC_ cells (selected to the same passage number in the absence of doxorubicin) are isogenic, since the vast majority of genes (92.6%) differed in expression by < 2-fold between the two cell lines [see statistical analysis of microarray (SAM) plots in Figure 1]. This suggests that observed differences in gene expression are likely related to the acquisition of doxorubicin resistance and not simply a selection for a rare, unrelated cell type within the cell population.

**Figure 2:**
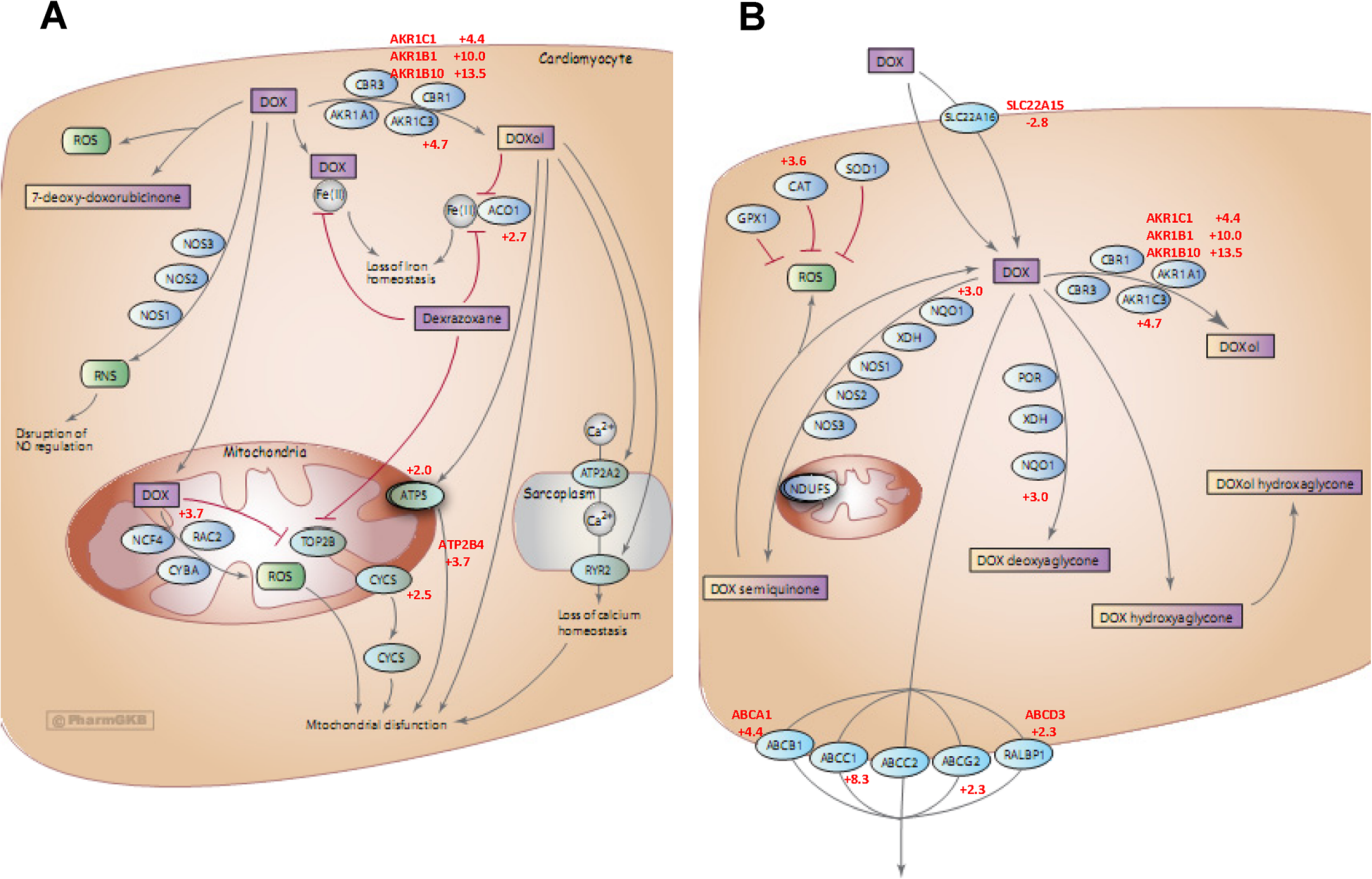
**PharmGKB doxorubicin pharmacodynamic and pharmacokinetic pathways.** Doxorubicin metabolic pathways were retrieved from the PharmGKB website (http://www.pharmgkb.org/drug/PA449412#tabview=tab4&subtab=33), combined, and used for further analysis. All diagrams are copyright PharmGKB. Used with permission from PharmGKB and Stanford University. (**A**) pharmacodynamics (**B**) Pharmacokinetics.

In examining the identities of genes exhibiting the greatest changes in expression upon acquisition of doxorubicin resistance, a number of these genes play a role in doxorubicin metabolism. Consequently, we assessed the extent of “over-representation” of doxorubicin metabolism genes by comparing the names of differentially expressed genes in the microarray “hit list” with those listed in a curated list of genes associated with doxorubicin pharmacokinetics or pharmacodynamics in tumour cells and cardiomyocytes available on the Pharmacogenetics Knowledge Base (PharmGKB) [16]. This list can be found at the url: http://www.pharmgkb.org/drug/PA449412#tabview=tab5&subtab=33 and is depicted in Additional file 1: Table S1. Figure 2 shows two pathway diagrams available through the PharmGKB website that document the different proteins that impact on the uptake, metabolism, and efflux of doxorubicin in cardiomyocytes (A) and tumour cells (B). A comparison of a list of these proteins (Additional file 1: Table 1) with the list of genes significantly changed by ≥ 2-fold in doxorubicin-resistant cells in the above microarray experiment (p ≤ 0.01) revealed that doxorubicin pharmacokinetic and pharmacodynamic genes are highly over-represented in the list of differentially expressed genes. Identical genes or genes having the same family name on both lists are depicted in bold, with the fold increase (+) or decrease (−) in expression in the microarray experiment listed beside each gene. Additional file 2: Table S2 depicts the results of our over-representation analysis. At a false discovery rate of 0.01, 8 of the 46 genes listed in the doxorubicin pharmacokinetics/ pharmacodynamics pathways (17%) were direct matches and 20 or 43% were partial matches (same gene family). The p value for significance of this over-representation relative to randomly selected genes was 0.05 for identical matches and < 0.0001 for either identical or partial matches. Since these genes directly affect the uptake, efflux, metabolism or cytotoxicity of doxorubicin, they have a strong potential to play a role in doxorubicin resistance. The identities of these genes provide a compelling view of the various mechanisms that likely play a role in the acquisition of doxorubicin resistance in breast tumour cells *in vitro* (see discussion).

### Several AKRs are over-expressed in MCF-7_DOX2-12_ cells

As previously demonstrated using a much smaller microarray platform (1720 genes) [17], the “1C” family of AKRs was observed to be over-expressed upon acquisition of doxorubicin resistance. Moreover, as shown in Additional file 1: Table S1, a variety of AKR family members were among the most differentially expressed genes upon acquisition of doxorubicin resistance in MCF-7 cells. In these microarray studies, AKR1B1, AKR1B10, AKR1C1, and AKR1C3 all had strongly elevated expression (10.0-, 13.4-, 4.45-, and 4.71-fold, respectively). As stated previously, the product of the AKR family of genes facilitates the conversion of doxorubicin to doxorubicinol [18]. Such a strong overexpression of multiple AKR transcripts in MCF-7_DOX2-12_ cells suggests that the AKRs may play a major role in doxorubicin resistance.

**Figure 3:**
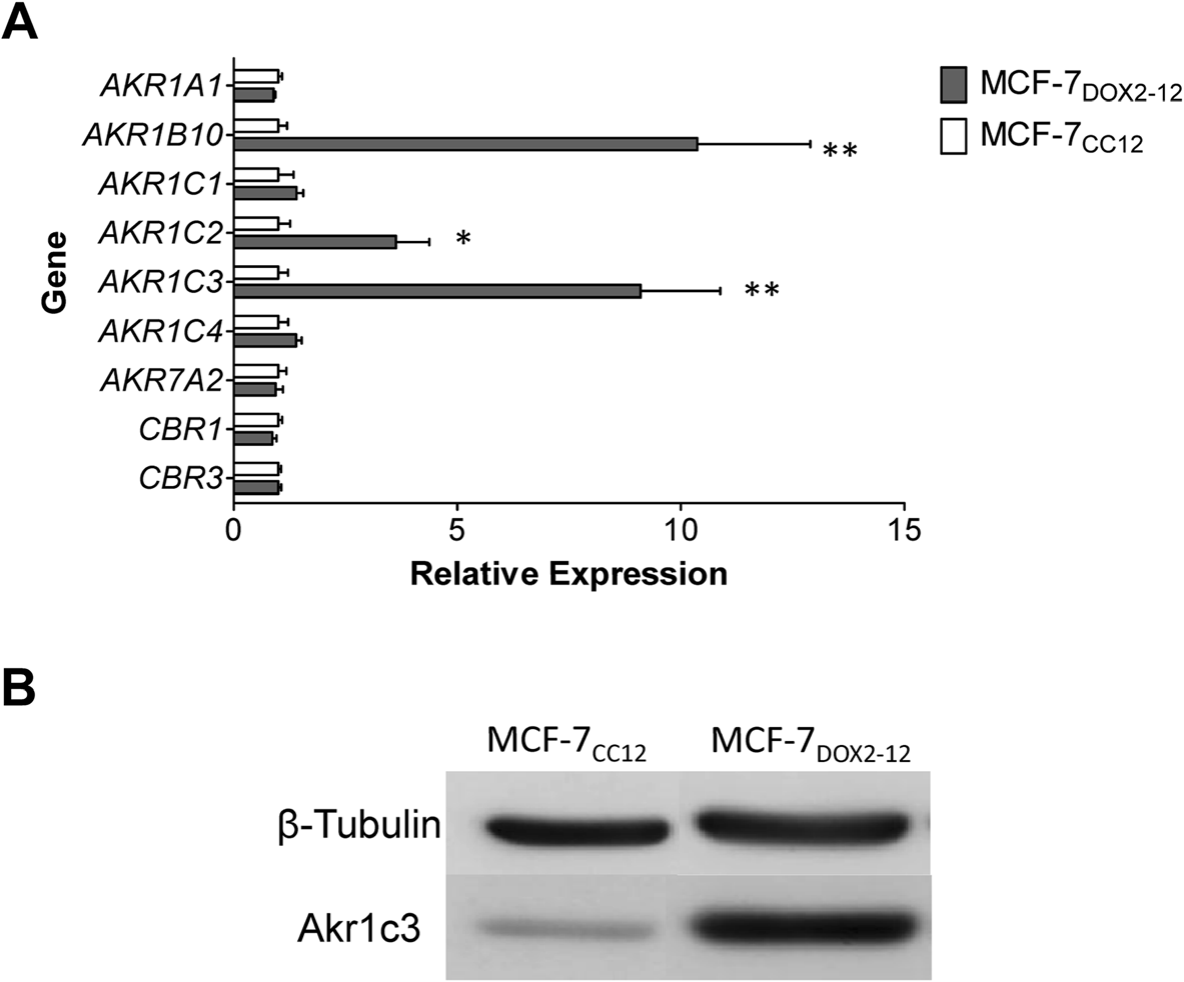
**Gene and protein expression levels of doxorubicin to doxorubicinol metabolizing enzymes.** (**A**) Relative changes in expression between MCF-7_CC12_ and MCF-7_DOX2-12_ cells were assessed by RTqPCR. Each sample was normalized first to RPS28 for loading and then to the average expression of MCF-7_CC12_ cells to determine fold change. * p ≤ 0.05, ** p ≤ 0.01. (**B**) Western blot analysis of AKR1C3 expression in MCF-7_DOX2-12_ and MCF-7_CC12_ whole cell lysates.

Given that AKR “1C” isoforms are highly conserved amongst each other [19] and given that, by BLAST analysis, the probes on the Agilent 4X44K arrays could not distinguish between the four 1C transcripts, we designed isoform-specific primers for reverse transcription quantitative polymerase chain reaction (RTqPCR) experiments in order to accurately quantify the levels of expression of these transcripts. Similarly, since substantial elevations in the highly conserved AKR “1B” isoforms were observed by microarray analysis with AKR1B probes that are not isoform-specific, we also designed isoform-specific RTqPCR primers to accurately quantify transcript levels for the two AKR 1B isoforms identified by microarray analysis. Finally, since the carbonyl reductases (CBRs), like the AKRs, can also play a role in the conversion of doxorubicin to doxorubicinol [8,20], we designed isoform-specific primers to quantify levels of transcripts for two CBR isoforms. The data from these RTqPCR experiments (Figure 3A) revealed that only the *AKR1C2*, *AKR1C3* and *AKR1B10* transcripts were significantly over-expressed in MCF-7_DOX2-12_ cells compared to MCF-7_CC12_ cells (3.6-, 9.1-, and 10.4-fold, respectively). *CBR1* and *CBR3* transcripts were not differentially expressed in the doxorubicin-resistant cells. As *AKR1C3* exhibited one of the highest changes in expression, and since Akr1c3 has been shown to efficiently convert doxorubicin to doxorubicinol [21], we also assessed the expression of Akr1c3 protein in the cell lines. As shown in Figure 3B, immunoblotting experiments confirmed the considerably higher expression of Akr1c3 in MCF-7_DOX2-12_ cells relative to MCF-7_CC12_ cells.

### Doxorubicinol is 1 million-fold less cytotoxic than doxorubicin in MCF-7 cells

**Figure 4:**
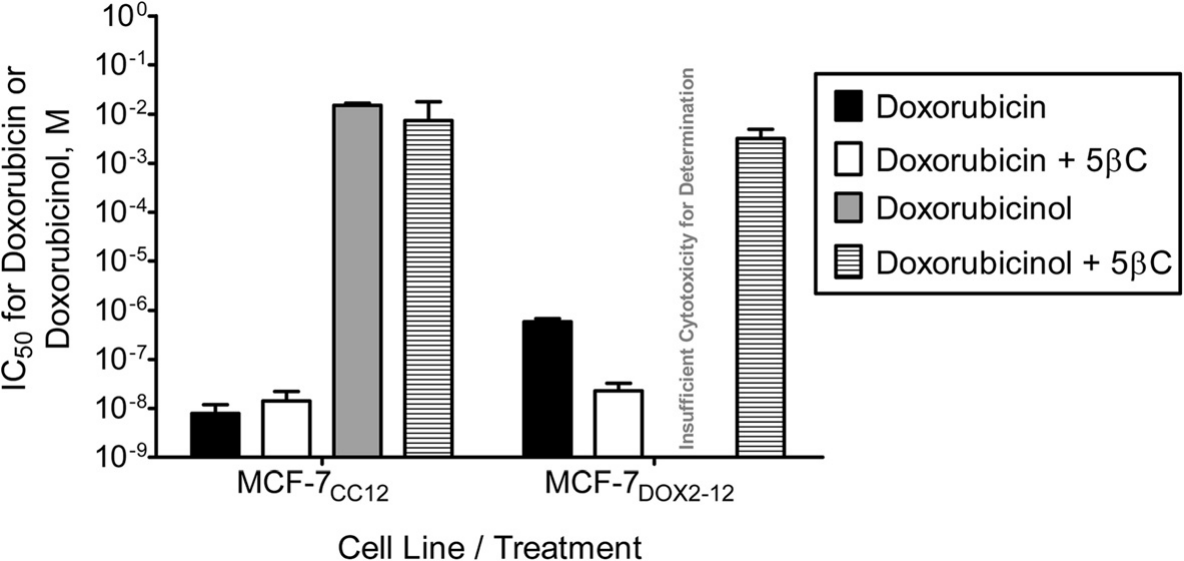
**Doxorubicin and doxorubicinol cytotoxicity in MCF-7**_**CC12**_**and MCF-7**_**DOX2-12**_**cells upon treatment with or without 5β-cholanic acid*.*** MCF-7_CC12_ and MCF-7_DOX2-12_ cells were assessed for their sensitivity to doxorubicin and doxorubicinol in the presence or absence of the AKR1B10, AKR1C2, and AKR1C3 inhibitor 5β-cholanic acid using a clonogenic assay.

Although it has been previously reported that doxorubicinol is 20 to 27 times less cytotoxic than doxorubicin in fibroblasts or pancreatic tumour cells [22,23], we also wanted to assess in this study the relative sensitivity of MCF-7_DOX2-12_ and MCF-7_CC12_ cells to doxorubicin and doxorubicinol. As shown in Figure 4, the concentration of doxorubicin required to reduce the number of colonies formed in a clonogenic assay by half (the IC_50_) was 7.8 ± 4.0 nM and 580 ± 91 nM for MCF-7_CC12_ and MCF-7_DOX2-12_ cells, respectively, indicating a 74-fold resistance to doxorobucin in MCF-7_DOX2-12_ cells. In contrast, the IC_50_ of doxorubicinol for the MCF-7_CC12_ cell line was 15 ± 1.6 mM, indicating a reduced cytotoxicity for the metabolite of over 6 orders of magnitude! Interestingly, the cytotoxicity of doxorubicinol was even less in MCF-7_DOX2-12_ cells. In fact, we could not achieve sufficient cytotoxcity to compute an IC_50_ value. Consequently, in our study, doxorubicinol cytotoxicity in a clonogenic assay was dramatically less than doxorubicin, suggesting that the conversion of doxorubicin to doxorubicinol by AKRs or CBRs would essentially eliminate its cytotoxicity in breast tumour cells in culture.

### 5β-cholanic acid restores sensitivity of MCF-7_DOX2-12_ cells to doxorubicin

As illustrated in Figure 4, treatment of MCF-7_DOX2-12_ cells with both doxorubicin and 5β-cholanic acid, a potent inhibitor of AKR1B10 [24], AKR1C2, and AKR1C3 activity [25], almost fully restored doxorubicin sensitivity to that of MCF-7_CC12_ cells (IC_50_ of 22.9 ± 9 nM). In contrast, treatment of MCF-7_CC12_ cells with 5β-cholanic acid and doxorubicin had little effect on doxorubicin sensitivity (IC_50_ of 14.1 ± 8.0 nM), suggesting insufficient AKR activity in these cells to affect doxorubicin sensitivity. Addition of 5β-cholanic acid had no effect on sensitivity of MCF-7_CC12_ cells to doxorubicinol (IC_50_ of 7.5 ± 10.6 mM). However, addition of 5β-cholanic acid to MCF-7_DOX2-12_ cells did appear to increase their sensitivity to doxorubicinol to a barely detectable range (IC_50_ of 3.2 ± 1.7 mM), suggesting a possible ability of the inhibitor to affect further metabolism of doxorubicinol in doxorubicin-resistant cells.

### Restoration of doxorubicin sensitivity is accompanied by restored nuclear localization in MCF-7_DOX2-12_ cells

**Figure 5:**
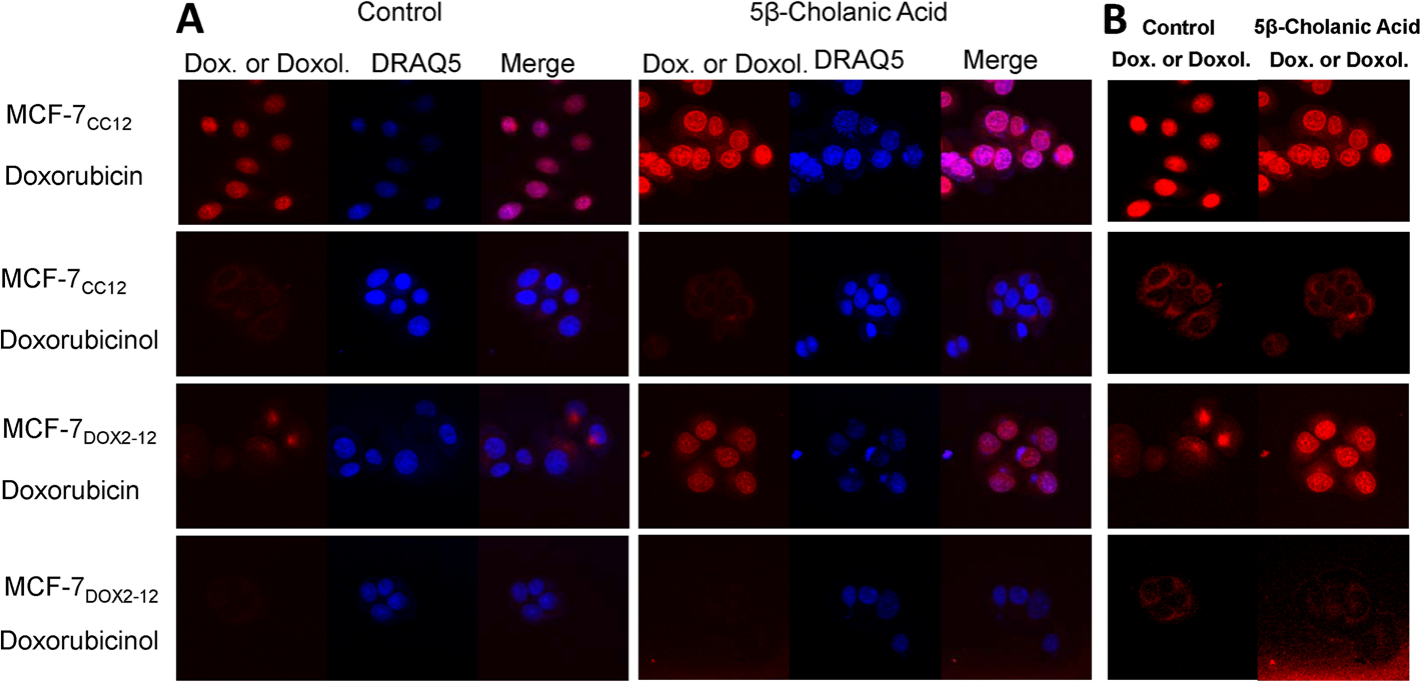
**Intracellular localization of doxorubicin and doxorubicinol in MCF-7**_**CC12**_**and MCF-7**_**DOX2-12**_**cells upon treatment with or without 5β-cholanic acid.** Cells were treated for 24 h with doxorubicin or doxorubicinol (red) in the presence or absence of the AKR1C2, AKR1C3, and AKR1B10 inhibitor 5β-cholanic acid. After treatment for 24 h, the nuclear dye DRAQ5 (blue) was added to the media as a counterstain for 15 minutes, and then coverslips were mounted and imaged by confocal fluorescence microscopy.

Since doxorubicin is a fluorescent, DNA-binding topoisomerase II inhibitor [1,2], it localizes to the nucleus in tumour cells. Drug localization can be effectively monitored by incubating cells with doxorubicin, and removing extracellular drug by extensive washing of the cells, followed by confocal laser scanning fluorescence microscopy [26]. We used this approach to visualize the location of doxorubicin (red fluorescence) in MCF-7_CC12_ and MCF-7_DOX2-12_ cells in the presence of DRAQ5, a highly cell-permeable DNA-binding dye that fluoresces into the infra-red region of the electromagnetic spectrum (shown as pseudo-blue fluorescence). As shown in Figure 5A, we found that fluorescence of 0.5 μM doxorubicin localized to nuclei in MCF-7_CC12_ cells, as expected (Figure 5). There was strong co-localization of doxorubicin and DRAQ5 fluorescence (bright purple fluorescence). In MCF-7_DOX2-12_ cells, however, greatly reduced doxorubicin fluorescence was observed, likely due to the reduced uptake of doxorubicin into these cells, as we previously reported [27]. In addition, the little fluorescence that was visible was extra nuclear (as visualized by a lack of co-localization with DRAQ5 staining), and this fluorescence was clustered in the perinuclear region. These observations are consistent with those pre-viously reported by Coley and colleagues for other doxorubicin-resistant cell lines [26]. In previous experiments, we observed that doxorubicin fluorescence in MCF-7_DOX2-12_ cells co-localized with Lysotracker™ [17] but not Mitotracker™ (Life Technologies, Burlington, VT) staining (data not shown), suggesting that the drug was sequestered in lysosomes and not bound to mitochondrial DNA. The inability of doxorubicin to reach its target (nuclear DNA) can clearly account for the reduced cytotoxicity of doxorubicin observed in MCF-7_DOX2-12_ cells. However, it is unclear whether the perinculear fluorescence exhibited in MCF-7_DOX2-12_ cells was from doxorubicin or perhaps a metabolite of doxorubicin that retains its fluroescence, such as doxorubicinol. As shown in Figure 5A, when identical experiments were performed with the equally fluorescent doxorubicinol, intracellular fluorescence was even weaker for MCF-7_DOX2-12_ cells. This may reflect a reduced and greatly reduced ability of doxorubicinol to enter MCF-7_CC12_ and MCF-7_DOX2-12_ cells, respectively. When microscope settings were adjusted to improve detection of these weak signals (Figure 5B), it was clear that doxorubicinol, unlike doxorubicin, localized outside of the nucleus in both cell lines, suggesting that the metabolite cannot reach or bind its target. This raises the prospect that some of the extra nuclear doxorubicin in MCF-7_DOX2-12_ cells may, in fact, be doxorubicinol or another fluorescent doxorubicin metabolite. However, the doxorubicin fluorescence in MCF-7_DOX2-12_ cells is much more concentrated in the perinuclear region and not as diffuse as doxorubicinol, suggesting the drug and its metabolite occupy distinct locations within cells.

We then assessed whether co-treatment of cells with 5β-cholanic acid altered doxorubicin or doxorubicinol localization (Figure 5). Interestingly, 200 μM 5β-cholanic acid was able to completely restore doxorubicin localization to the nucleus of MCF-7_DOX2-12_ cells, suggesting that the conversion of doxorubicin to doxorubicinol does alter the drug’s ability to reach or bind its target. The same concentration of 5β-cholanic acid, however, had no effect on doxorubicinol localization in MCF-7_CC12_ and MCF-7_DOX2-12_ cells.

### Doxorubicinol fails to accumulate in MCF-7_CC12_ and MCF-7_DOX2-12_ cells

**Figure 6:**
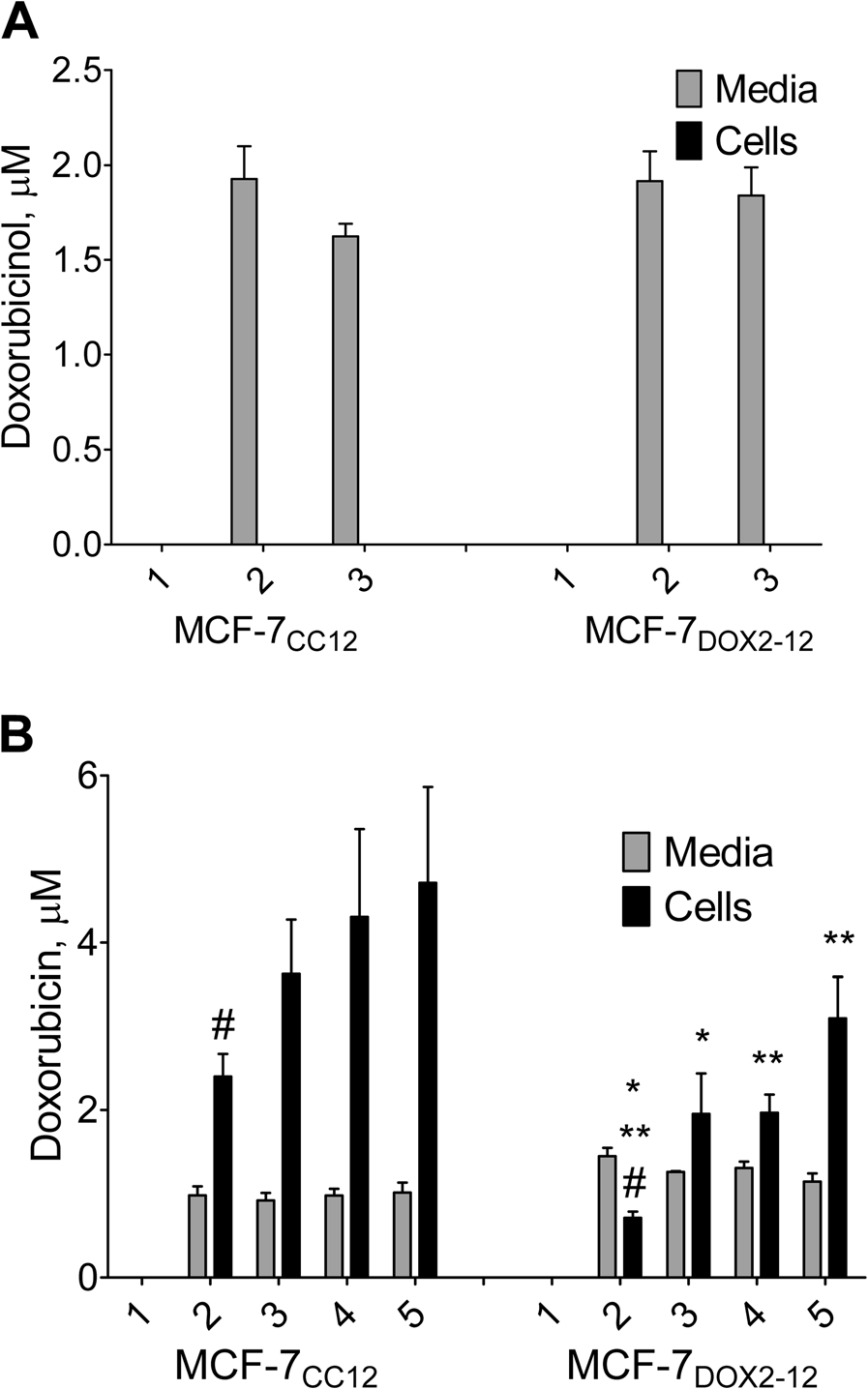
**Intracellular levels of doxorubicin or doxorubicinol in the presence or absence of 5β-cholanic acid or cyclosporine A, measured by HPLC.** MCF-7_CC12_ and MCF-_7DOX2-12_ cells were treated for 24 h with 0.5uM doxorubicin or doxorubicinol, 200uM 5β-cholanic acid, and/or 5uM cyclosporine A. After treatment, cellular and media extracts were prepared and assessed for doxorubicin or doxorubicinol content by HPLC: (**A**) Treatment with doxorubicinol and 5β-cholanic acid: 1 = no treatment; 2 = 0.5uM doxorubicinol; 3 = 0.5uM doxorubicinol and 200uM 5β-cholanic acid, (**B**) Treatment with doxorubicin, 5β-cholanic acid, and/or cyclosporine A: 1 = no treatment; 2 = 0.5uM doxorubicin; 3 = 0.5uM doxorubicin + 200uM 5β-cholanic acid; 4 = 0.5uM doxorubicin + 5uM cyclosporine A; 5 = 0.5uM doxorubicin + 200uM 5β-cholanic acid + 5uM cyclosporine A. The symbol # represents differences between MCF-7_CC12_ and MCF-7_DOX2-12_ cells (treatment 2) with a p value ≤ 0.01. The symbols * and ** represent significant differences between treatments at p values of ≤ 0.05 and ≤ 0.01, respectively.

After incubation with 0.5 μM doxorubicin, we used high performance liquid chromatography to assess the level of doxorubicin and doxorubicinol in MCF-7_CC12_ and MCF-7_DOX2-12_ cells and in the medium in which they grew. As shown in Figure 6, there was no detectable doxorubicinol in doxorubicin-treated MCF-7_CC12_ cells or in their cell culture medium, suggesting minimal expression of AKRs or CBRs. However, we surprisingly did not detect any doxorubicinol in MCF-7_DOX2-12_ cells or their medium, despite their higher levels of expression of AKR isoforms in the cells. Added doxorubicinol to cells could be extracted and quantified in the medium and in cells (Figure 6A), suggesting that the negative result was not due to an inability of the method to detect doxorubicinol. Treatment of either cell line with 5β-cholanic acid did not affect the intracellular level of doxorubicinol or the levels of doxorubicinol in the media.

### Intracellular levels of doxorubicin are significantly altered upon treatment of MCF-7_DOX2-12_ cells with 5β-cholanic acid and/or cyclosporine A

Treatment of MCF-7_CC12_ cells with 5β-cholanic acid and the pan ABC transporter inhibitor cyclosporine A increased cellular doxorubicin content by 51% and 80%, respectively (Figure 6B). Addition of both agents increased doxorubicin content to almost twice that of untreated cells, but none of the above differences in doxorubicin content were considered statistically significant. In contrast, 5β-cholanic acid or cyclosporine A significantly increased doxorubicin content in MCF-7_DOX2-12_ cells by 2.8-fold (Figure 6B). Treatment of MCF-7_DOX2-12_ cells with both 5β-cholanic acid and cyclosporine A increased cellular doxorubicin content to levels 4.4-fold higher than untreated cells (Figure 6B). These differences relative to untreated cells were found to be highly significant, and are likely due to the increased expression of AKRs [17] and ABC drug transporters known to be over-expressed in MCF-7_DOX2-12_ cells, including Abcc1 [27].

### Doxorubicinol binds to DNA with lower affinity than doxorubicin

**Figure 7:**
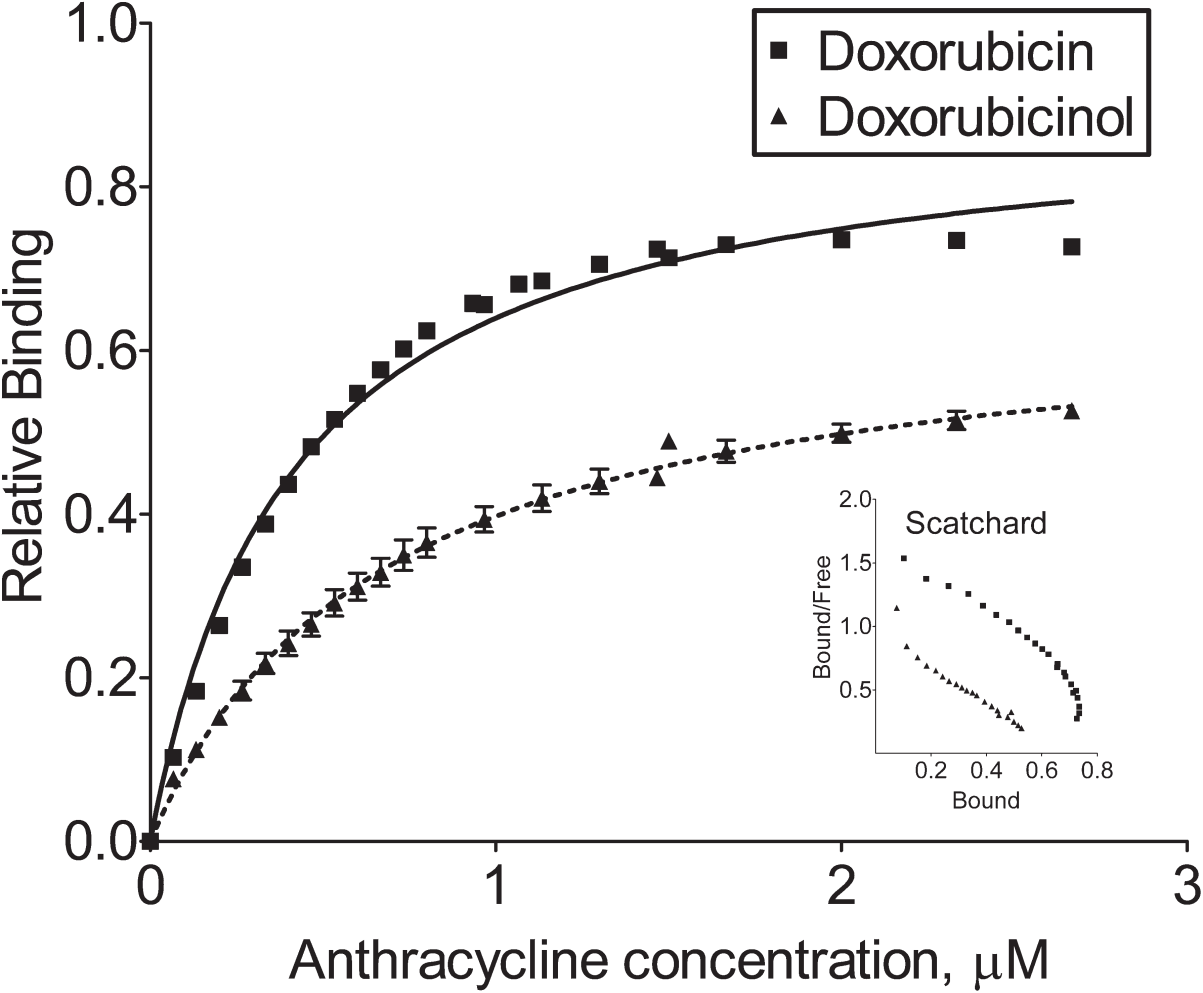
**Relative DNA binding affinity of doxorubicin and doxorubicinol measured by a FID assay.** Relative DNA binding affinity of doxorubicin and doxorubicinol was measured by adding consecutive aliquots of either doxorubicin or doxorubicinol to ethidium bromide-saturated salmon sperm DNA. Addition of the doxorubicin or doxorubicinol displaced the ethidium bromide and resulted in a progressive net decrease in fluorescence at excitation 545 nm and emission 595 nm.

We theorized that doxorubicinol does not localize to the nuclei of MCF-7_CC12_ and MCF-7_DOX2-12_ cells because the hydroxylation of doxorubicin reduces its affinity for DNA. To test this hypothesis, we compared the DNA binding parameters of doxorubicin and doxorubicinol using a binding displacement assay described in Methods. As shown in Figure 7 and Additional file 3: Table S3, both B_max_ and K_app_ were substantially different between doxorubicinol (0.667 ± 0.013 and 0.679 ± 0.034 μM, respectively) and doxorubicin (0.903 ± 0.012 and 0.412 ± 0.017 μM, respectively), suggesting that, on a molar basis, doxorubicinol binds to DNA with a much lower affinity and capacity than doxorubicin.

## Discussion

### Use of the binomial statistic to interpret the significance of pathways in gene expression data

DNA microarray, high throughput quantitative PCR, and other gene profiling approaches have been highly useful in identifying differences in gene expression between cells or tumours responding to chemotherapy agents and those that do not. Unfortunately, the false discovery rate for such approaches is quite high, largely due to the identification of a large number of “passenger genes” unrelated to drug response. A wide variety of pathway analysis tools exist today, some manually curated, and some created primarily through machine learning. PharmGKB [28], Ariadne Pathway Studio [29], Reactome [30], Ingenuity Pathway Analysis (http://www.ingenuity.com/), GenMAPP [31], and DAVID [32], are examples of available tools which can be used to map changes in gene expression to alterations in biochemical pathways. The difficulty with this approach is the sheer size of the data sets, the large number of documented pathways, and the complex statistics required to determine the significance of findings. In this study we elected to use a simple model to examine the biology of doxorubicin resistance, namely looking for “over-representation” of doxorubicin pharmacokinetic and pharmacodynamic genes in datasets of genes having altered expression in doxorubicin resistance.

In order to assess the feasibility of this approach and to survey the broadest number of genes, we used Agilent full genome microarrays containing 27,958 Entrez gene probes, unlike our previous study of only 1720 gene probes [17]. This approach helped to uncover a number of AKRs induced during selection for doxorubicin resistance, including AKR1C1, AKR1B1, AKR1B10, and AKR1C3. Their expression was elevated between 4.5- and 13.4-fold (Additional file 1: Table S1). Given that the probes for these AKRs on the Agilent microarrays were not isoform-specific, we used RTqPCR with isoform-specific primers (Table 4) to determine that, upon selection for doxorubicin resistance, transcripts for AKR1C2, AKR1C3, and AKR1B10 were overexpressed 3.6-, 9.1-, and 10.4-fold, respectively (Figure 3). In addition to the AKRs, other over-represented genes (Figure 2) provide further insight into other proteins that likely contribute to doxorubicin resistance. For example, *NQO1* codes for NAD(P)H dehydrogenase quinone 1, which plays a role in converting doxorubicin to doxorubicin deoxyaglycone or to doxorubicin semiquinone (Figure 2). Its 3-fold increase in expression might therefore increase the conversion of doxorubicin to these metabolites as well. Transcripts for the drug efflux pump Abcc1 were also upregulated 8.3-fold, as well as transcripts for other ATP-binding cassette (ABC) transporters such as Abcd3, Abcg2, and Abca1. In addition, a gene (*SLC22A15*) homologous to the solute carrier protein Slc22a16 (which promotes doxorubicin uptake into cells [33]) was found to be down regulated by 2.8-fold. The combined changes in the expression of ABC transporters and solute carrier proteins would be expected to reduce doxorubicin accumulation into cells. The gene for catalase (*CAT*) was found to be upregulated 3.6-fold in MCF-7_DOX2-12_ cells. Since its gene product helps protect cells from oxidative damage by reactive oxygen species [34], its elevated expression would protect cells from reactive oxygen species known to be generated by doxorubicin. Genes associated with the cardiotoxicity of doxorubicin (through negative effects on mitochondrial function when converted to doxorubicinol) also have altered expression in breast tumour cells upon selection for doxorubicin resistance, including ACO1, ATPS, CYCS, and ATP2B4 (Figure 2 and Additional file 1: Table S1).

Of the above-described changes in gene expression, the greatest were for the AKRs. Evidence provided in this study supports their substantial role in doxorubicin resistance in tumour cells *in vitro*, and possibly in the tumours of cancer patients. While many of the changes in gene expression identified in our microarray study likely play a *bona fide* role in doxorubicin resistance (given their roles in cells), some of the identified genes may not be the “drivers” of drug resistance, but change expression through the altered expression of the driver genes.

### Role of the AKRs in resistance to doxorubicin

A role for AKRs in xenobiotic and anthracycline metabolism has already been well established in the literature [21,35-37]. We also published previously that aldo-keto reductases (AKRs) are overexpressed upon acquisition of anthracycline resistance, that doxorubicin localization to the nucleus is altered in doxorubicin-resistant cells, and that inhibition of AKRs restores doxorubicin sensitivity in doxorubicin-resistant cells [17]. However, the current study significantly extends these observations in many respects. For example, it reveals that the expression of other members of the AKR family is elevated as breast tumour cells acquire resistance to doxorubicin. This would further increase the production of doxorubicinol and its possible conversion to other downstream metabolites. Moreover, our study provides a detailed comparison between doxorubicin and doxorubicinol in terms of their cytotoxicity, subcellular localization, and DNA binding activity.

Interestingly, despite having identical fluorescence capacities, cellular levels of doxorubicinol in both MCF-7_CC12_ and MCF-7_DOX2-12_ cells was considerably lower than that of doxorubicin (as measured by cellular fluorescence intensity after drug administration and washing away free drug not taken up by cells). This decreased doxorubicinol uptake may be because hydroxylated doxorubicin is more polar and less able to traverse the hydrophobic plasma membrane. Moreover, even if the confocal microscope settings are modified to allow greater sensitivity to detect cellular doxorubicinol, doxorubicinol was found not to be localized to the nucleus in both MCF-7_CC12_ and MCF-7_DOX2-12_ cells. This indicates that the differential localization of doxorubicin between MCF-7_CC12_ and MCF-7_DOX2-12_ cells may be due to the strongly elevated conversion of doxorubicin to doxorubicinol (or other fluorescent metabolites) in MCF-7_DOX2-12_ cells. This may be why “doxorubicin” had an altered location in anthracycline-resistant cells in our previous study. The fluorescence observed in lysosomes may be that of doxorubicin, but also of doxorubicinol and other fluorescent doxorubicin metabolites. Consistent with this view, and not reported in our previous study, the administration of the AKR inhibitor 5β-cholanic acid significantly restored “doxorubicin” localization to the nucleus. More likely the inhibitor prevented doxorubicin conversion to doxorubicinol, permitting more doxorubicin to be retained within the nucleus.

What could account for the decreased localization of doxorubicin to the nucleus? We report in the current study that doxorubicinol has significantly lower ability to bind to DNA than doxorubicin (altered B_max_ and K_app_)_._ The conversion of doxorubicin to doxorubicinol by AKRs would result in reduced binding to DNA and hence less ability of the drug to remain associated with the nucleus. In our previous study, we did not differentiate between the cellular localization of doxorubicin and doxorubicinol.

One surprising finding in our study was the lack of detection of significant doxorubicinol in MCF-7_DOX2-12_ cells (Figure 6). This was despite the elevated expression of a number of AKRs in the cell line (Figure 3), which would be expected to covert doxorubicin to doxorubicinol. And yet, the addition of 5β-cholanic acid with doxorubicin increased the cellular content of doxorubicin (Figure 6), supporting the observation that 5β-cholanic acid is able to block the conversion of doxorubicin to doxorubicinol. What may account for the discrepancy in these points of view? One possibility is that 5β-cholanic acid blocks the efflux of doxorubicin by drug transporters (possibly Abcc1), thereby increasing the retention of doxorubicin in cells. One argument against this hypothesis is that both 5β-cholanic acid and cyclosporine A increased cellular doxorubicin content (Figure 6), the latter being a known inhibitor of Abcc1 function [38]. The combination of both agents increased cellular doxorubicin content further, suggesting that they were acting by distinct mechanisms. Moreover, unlike 5β-cholanic acid (Figure 4), addition of cyclosporine A had no effect on the cytotoxicity of doxorubicin in MCF-7_DOX2-12_ cells, as measured in a clonogenic assay [27]. Finally, another inhibitor of AKR catalytic activity with a structure very distinct from cyclosporine A (flufenamic acid) also restored doxorubicin cytotoxicity and nuclear localization in MCF-7_DOX2-12_ cells (data not shown). This suggests that it is the ability of these agents to inhibit AKR activity that is responsible for the restoration of drug cytotoxicity. An alternative argument is that the doxorubicinol, once formed, is further metabolized, such that the metabolite is not retained in the method used to extract cellular doxorubicin and doxorubicinol for HPLC-based measurements. Thus, doxorubicinol would not be seen to accumulate in MCF-7_DOX2-12_ cells.

Despite the ability of both cyclosporin A and 5β-cholanic acid to increase cellular doxorubicin content in MCF-7_DOX2-12_ cells, why was only the latter agent able to appreciably restore doxorubicin cytotoxicity? Increasing the cellular content of doxorubicin by the cyclosporine-mediated reduction of drug efflux may not sufficiently increase its cytotoxicity if the additional cellular doxorubicin is rapidly converted to doxorubicinol by the elevated expression of AKRs and/or if the additional doxorubicin is sequestered into lysosomes. In contrast, AKR inhibition may block all conversion of doxorubicin to doxorubicinol, such that any drug entering the cell remains as doxorubicin and is able to rapidly reach the nucleus, before being sequestered.

## Conclusions

Using a full genome approach, this study provides important new insight into pharmacokinetic and pharmacodynamic pathways that are altered upon selection of cells for resistance to doxorubicin. In addition to our previously reported finding of increased expression of the AKR 1C isoforms [17], the current study reveals other changes in gene expression that would be expected to affect the cytotoxicity of doxorubicin. This includes genes that may: decrease uptake of doxorubicin (SLC22A15), enhance efflux of doxorubicin (ABCC1, ABCG2, ABCD3, ABCA1), enhance conversion of doxorubicin to doxorubicinol (AKR1B10, AKR1B1), doxorubicin deoxyaglycone or doxorubicin semiquinone (NQO1), and inhibit the ability of doxorubicin to damage tumour cells through the generation of reactive oxygen species (CAT). Moreover, this study provides an in-depth comparison of the biochemical properties of doxorubicin versus doxorubicinol. While the former is highly cytotoxic, has high DNA binding affinity, and localizes to the nucleus in wildtype breast tumour cells, doxorubicinol is over a million times less cytotoxoic, has significantly reduced DNA binding activity, and is retained in the cytoplasm or lysosomes of cells. We also show that the administration of AKR inhibitors with doxorubicin in MCF-7_DOX2_ cells substantially restores both drug localization to the nucleus and drug cytotoxicity. Interestingly, doxorubicinol is highly cardiotoxic, and it is believed that doxorubicinol is responsible for the cardiotoxicity associated with doxorubicin chemotherapy [39,40]. Since the AKR inhibitor 5β-cholanic acid is a well-tolerated naturally occurring bile acid in humans, and since flufenamic acid has been used in clinical trials with manageable toxicities [41], there may be significant value in conducting clinical trials in which either 5β-cholanic acid or flufenamic acid are co-administered with doxorubicin during chemotherapy. Results in this study would suggest that these AKR inhibitors may increase tumour levels of doxorubicin and block cardiotoxicity induced by doxorubicin conversion to doxorubicinol. This may dramatically improve the therapeutic index of doxorubicin when administered to cancer patients and improve the duration of clinical response for this otherwise highly effective chemotherapy drug.

## Methods

### Supplies and reagents

Supplies and reagents used in this study came from a variety of sources. Unless otherwise noted, Sigma (St. Louis, MO) was the supplier.

### Cell culture

MCF-7 breast adenocarcinoma cells were obtained from the American Tissue Culture Collection (ATCC) (lot HTB-22) and selected for resistance to doxorubicin (Pfizer Pharmaceuticals, St. Laurent, QC) as previously described [27]. Briefly, doxorubicin-sensitive, wildtype MCF-7 cells were grown in progressively increasing concentrations of doxorubicin from 1000x below the IC_50_ for the drug in parental MCF-7 cells (dose 1) to its maximally tolerated dose (dose 12) in 1.5- or 3-fold increments, with retention of cells surviving the greater of the two doses. Cells selected for survival in the varying doses of doxorubicin were termed MCF-7_DOX2_ cells. A “co-cultured control” cell line was selected under identical conditions in the absence of drug (MCF-7_CC_ cells)_._ These cells served as a control to help identify changes in gene expression due to long-term cell culture. The highest dose level to which cells were selected are indicated in the subscript of the cell line name. For example, MCF-7_DOX2-12_ cells refers to cells selected to the 12^th^ dose level of doxorubicin. The 2 in the subscript is to prevent confusion with a previously isolated doxorubicin-resistant cell line in our laboratory (MCF-7_DOX_). All cells used in this study were selected to dose level 12 (0.1 μM doxorubicin). Cells were grown in high-glucose DMEM medium (Fisher Scientific, Nepean, ON) supplemented with penicillin-streptomycin (Fisher) and 10% fetal bovine serum (Fisher) in 75 cm^2^ tissue culture flasks (Sarstedt Canada, Montreal, QC), unless otherwise noted. Cells were maintained at 37°C in air supplemented with 5% CO_2_ in a humidified environment. Cells were passaged weekly, with a medium change once between passages. Drug-resistant cells were maintained in medium containing doxorubicin at their selection dose.

### Microarray analysis

Changes in gene expression between MCF-7_CC12_ and MCF-7_DOX2-12_ cells were identified by microarray analysis using Agilent 4x44k whole human genome arrays (product number G4112F; Agilent Technologies, Mississauga, ON). These arrays enabled us to determine the level of expression of 27,958 human Entrez genes (close to the entire genome). Five hundred ng of total RNA, isolated with a Qiagen RNeasy kit (Mississauga, ON), was used for each sample. The RNA was then labeled with Cy3 or Cy5 using an Agilent Quick Amp labeling kit (Product #5190-0444). Hybridization was performed as per the manufacturer’s protocol. Experiments were repeated using multiple batches of labeled RNA, with both forward and reverse-labeling to account for dye bias, for a total of 16 two-colour arrays. The microarrays were scanned, and feature extraction and background intensity corrections were performed with Agilent software (v.10.7.3.1). Using Partek Genomics suite (St. Louis, MO) to perform a 4 way ANOVA using the Method of Moments [42], a list of genes significantly over- or under-expressed in MCF-7_DOX2-12_ cells relative to MCF-7_CC12_ cells. The false discovery rate was set at 0.01, with only genes changing expression by ≥ 2-fold being noted. The four variables assessed in the 4 way ANOVA were the cell line (MCF-7_CC12_ cells versus MCF-7_DOX2-12_ cells), the dye used (Cy3 versus Cy5), the experimental batch of arrays (to address batch effects) and the arrays themselves to address random effects. The input file was the data from all 16 two-colour arrays comparing gene expression between MCF-7_DOX2-12_ and MCF-7_CC12_ cells. The model used was: Yijklm = μ + Cell line (i) + Dye (j) + Exp batch (k) + arrays (random effect) (kl) + εijklm, where Yijklm represents the mth observation on the ith Cell line jth Dye kth Exp batch lth arrays, μ is the common effect for the whole experiment, εijklm represents the random error present in the mth observation, on the ith Cell line, jth Dye, kth Exp batch, lth arrays. The errors εijklm were assumed to be normally and independently distributed , with mean 0 and standard deviation δ for all measurements. Arrays and Exp batch were considered random effects. Normalized expression was transformed to the base 2.0, with p values reported for significance of differences in the expression of each gene. The output of the analysis was a p value for the significance of the observed fold change in expression of a particular gene and for the mean ratio of expression of a particular gene between the two cell lines. The microarray data was deposited in the NCBI Gene Expression Omnibus (GEO) database, accession number GSE27254) in accordance with MIAME standards [15]. The url to access this data is: http://www.ncbi.nlm.nih.gov/geo/query/acc.cgi?token=dbezngycywquuhm&acc=GSE27254.

To confirm the isogenic nature of the two cell lines, a Significance Analysis of Microarrays (SAM) plot was created using the freely-available microarray software TM4 suite [43], with the following workflow:

(1) Agilent raw data was converted to .MEV format using TIGR Express Converter v2.1.

(2) The MEV files were then normalized using TIGR MIDAS v2.22 and a LOWESS normalization filter with flip-dye consistency checking where appropriate.

(3) A one-class Significance Analysis of Microarrays (SAM) analysis was performed using TIGR MEV v4.6.1.

For pathway analysis, the list of significant differentially-expressed genes generated by Partek Genomics Suite was compared to a curated list of genes, transcripts or proteins shown to be involved in doxorubicin pharmacokinetics and pharmacodynamics in tumour cells or cardiomyocytes, available from the Pharmacogenomics Knowledge Base (PharmGKB) [16,28]. These lists were then compared in S-Plus (v8.0) using the binomial statistical test as previously described [14].

### RNA extraction, reverse transcription, and quantitative polymerase chain reaction

Given that the 1C and 1B AKR isoforms are highly conserved and that the probes for the 1C and 1B AKR transcripts on the Agilent 4X44 microarrays were not isoform-specific, we designed isoform-specific 1C and 1B primers to accurately quantify the levels of expression of the various 1C and 1B transcripts. These primers (Table 4) and isoform-specific primers for the carbonyl reductases (which also convert doxorubicin to doxorubicinol) were used in RTqPCR experiments. Total RNA was extracted from the MCF-7_CC12_ and MCF-7_DOX2-12_ cell lines using a Qiagen RNeasy kit (Mississauga, ON), reverse transcribed, and the cDNAs amplified using an ABI 7900HT quantitative PCR machine and SYBR Green 1 detection chemistry as described previously [44]. After RNA extraction and before reverse transcription, RNA was quantified and quality ensured using an Agilent Bioanalyzer 2100 RNA nano kit. 2 μg of RNA was then DNase I (Invitrogen) treated. For reverse transcription, either MMLV reverse transcriptase (Invitrogen, Burlington, ON) and an oligo-dT_20_ primer, or Superscript III reverse transcriptase (Invitrogen) and an AKR1C-specific reverse transcription primer were used, all according to the manufacturer’s protocol. The cDNA was then stored at −20°C until analysis via the ΔCT method. All experiments were performed according to MIQE standards [45]. Primers used for reverse transcription or to amplify specific cDNAs are described in Additional file 4: Table S4.

### Protein extraction and quantification

Ten cm plates of cells (cultured for 2 passages without drug to 80% confluence) were rinsed twice in Dulbecco’s PBS, removed of excess liquid using a pipette tip, and placed on ice. Three hundred to 1000 μl of RIPA buffer (10 mM Tris–HCl, 1% sodium deoxycholate, 0.1% SDS, 1% Triton X-100, 150 mM NaCl, pH 7.5) with added Complete™ protease inhibitors (Roche Diagnostics, Laval, QC) were added to each plate, and the plates scraped with a cell scraper. The resulting crude lysate was passed through a 21 gauge needle 5 times. The lysate was incubated on ice for 30 minutes and then centrifuged for 20 minutes at 13.2 × 10^3^ ×*g* at 4°C. The supernatant was retained and stored at −80°C. Protein concentrations in extracts were measured using a BCA protein quantification kit (Pierce Bioscience) using standard solutions of Bovine Serum Albumin (BSA).

### Western blotting

Forty μg of protein were diluted in 6x Laemelli loading buffer and loaded onto a 10% SDS-PAGE gel with a 4% stacking gel. The gel was also loaded with 5μL of Bio-Rad (Mississagua, ON) dual colour protein marker. The gels were subjected to electrophoresis in a Bio-Rad mini tetra system at 80 V for 30 minutes and then at 120 V for an additional 1 h. The gels were removed and the gel proteins transferred to nitrocellulose membranes (GE Healthcare, Baie d’Urfe QC) using a Bio-Rad semi-dry electroblotting apparatus for 1 h at 12v. Membranes were then stained with 0.5% Ponceau S in 1% acetic acid to confirm transfer efficiency and even protein loading. The membranes were blocked in 5% skim milk powder (Carnation) in 0.1% TBST (20 mM Tris–HCl, 150 mM NaCl, 0.05% Tween 20, pH 7.5) for 1 h at room temperature, and then incubated overnight at 4°C with an isoform specific mouse monoclonal anti-AKR1C3 antibody [46] (Sigma) at a 1:2,000 dilution in 5% skim milk powder in TBST. The membranes were then washed with TBST for 15 minutes, and incubated in HRP-conjugated goat anti-mouse secondary antibody (Santa Cruz Biotechnology Inc., Santa Cruz, CA) at a 1:10,000 dilution in 5% skim milk powder in TBST for 1 h at room temperature. Membranes were again washed in TBST for 15 minutes and subjected to 3 × 5 minute final washes in TBS before being covered in ECL solution (Santa Cruz Biotechnology Inc.) and imaged using a gel documentation system (Alpha Innotech) for 10 minutes. An identical procedure using a mouse monoclonal anti-β-tubulin antibody (Santa Cruz Biotechnology Inc.) at a 1:10,000 dilution was used to monitor β-tubulin levels in the extracts as a loading control.

### Confocal microscopy

Cells were plated on #1 coverslips placed in 6-well plates and allowed to adhere overnight before being treated. Treatment consisted of addition of 0.5 μM doxorubicin or doxorubicinol (Toronto Research Chemicals Inc., North York, ON), and either 200 μM 5β-cholanic acid (Steraloids, Newport, RI) or DMSO as a vehicle control. After the 24 h treatment, DRAQ5 (Biostatus, Leicestershire, UK) was added to the culture media for 15 minutes as a nuclear counterstain. The coverslips were rinsed gently in 3 sequential PBS washes and sealed onto standard microscope slides using clear nail polish. After the nail polish dried, cells were observed using a Zeiss LSM 510 META confocal laser scanning microscope using an argon-ion laser at a 488 nm wavelength band for excitation of doxorubicin and doxorubicinol and using a 560 nm long-pass filter to detect intrinsic fluorescence of doxorubicin and its metabolites. A 633 nm laser with a 650 nm long-pass filter was used to detect DRAQ5 fluorescence.

### High-performance liquid chromatography

Cells (8.0 × 10^6^ cells per 10 cm plate) were allowed to adhere overnight, after which they were treated with 0.5 μM doxorubicin or 0.5 μM doxorubicinol (with or without 5 μM cyclosporine A and/or 200 μM 5β-cholanic acid) for 24 h. After this time period, the media was decanted (with 0.5 mL reserved for HPLC analysis), and the plates were rinsed twice in PBS. One mL of a 0.2 M Na_2_HPO_4_ solution, pH 8.5, was added to the plates and the cells were scraped off of the plate. A 0.5 ml volume of the same solution was added to the 0.5 mL of reserved media. Each sample was then added to 4 mL of a 9:1 v/v chloroform:n-heptanol mixture in a polypropylene 15 mL centrifuge tube and shaken on a mixer for 20 minutes, after which the samples were centrifuged for 10 minutes at 2000× *g* at 20°C. The bottom organic layer was then aspirated from the tube using a glass 5 mL pipette and dispensed into a new 15 mL centrifuge tube containing 250uL of 0.1 M ortho-phosphoric acid. Each tube was then mixed on a vortex mixer for 30 seconds before being centrifuged for 2 minutes at 2000× *g*. The top 200μL of the upper aqueous layer was then removed and stored at −80 degrees Celsius for later analysis.

Separations were performed using a revised gradient elution based on a previously described isocratic method [17] on a Waters Alliance e2695 system with a Waters 2475 fluorescence detector set at 480 nm excitation and 560 nm emission. Chromatographic conditions were the following: column: YMC CN 25 × 5 mm column; Eluent A: 10 mM NaH_2_PO_4_ pH 4.0, Eluent B: HPLC grade CH_3_CN; flow rate: 1.0 mL/min. The gradient program was as follows: 0 min = 20% B 80% A, 10 min = 50% B 50% A, 11 to 24 min = 20% B 80% A. The slope for each gradient change was linear.

### DNA binding affinity assay

The relative DNA binding affinity of doxorubicin and doxorubicinol was determined by using a fluorescent intercalator displacement assay [47]. Briefly, a quartz cuvette was filled with 3 mL of Tris buffer (0.1 M Tris, 0.1 M NaCl, pH 8.0; approximating conditions inside the nucleus) to which 4.4 μM ethidium bromide was added. A fluorescence reading (excitation: 545 nm, emission: 590 nm) was taken using a Perkin Elmer LS-50 fluorimeter; this constituted the baseline reading. Pre-sheared salmon sperm DNA (8.8 μM in base pairs) was then added to the cuvette, incubated for 5 minutes, and again the fluorescence was determined; this constituted the maximal or 100% reading. Aliquots of doxorubicin or doxorubicinol (0.067 μM) were added to the cuvette, incubated for 5 minutes, and the corresponding reading recorded. The background reading was subtracted for each reading and then divided by the maximal reading to determine per cent of maximal binding. These data were fit to curves to determine K_app_ and B_max_ values.

### Measurement of drug sensitivity

Drug sensitivity was assessed using a variation [48] of the standard clonogenic assay [49]. Briefly, for each condition, 12 × 25 cm^2^ flasks were plated with 2.5 × 10^5^ cells and left to adhere overnight. The next day, each flask was treated with a different concentration of doxorubicin, decreasing in 3-fold increments, from 3.0 × 10^-6^ M to 5.13 × 10^-11^ M, with a final flask receiving no doxorubicin. After 24 h, cells were trypsinized, pelleted, and resuspended in 300uL of medium which was then combined with 2.7 mL of methyl cellulose growth medium [2.6% methyl cellulose, (Shin-Etsu) and 30% FBS in IMDM (Princess Margaret Hospital)]. After being mixed thoroughly, the suspension was allowed to settle for 30 minutes before 1.2 ml of cells were introduced into 6-well tissue culture plates. Plates were incubated for 2 weeks and then 10 randomly selected fields in each well were counted at 40x magnification.

### Statistical analyses

Graphpad Prism (v5.0) was used for all statistical tests unless otherwise noted. Differences between treatment means were assessed using either a Student’s unpaired t-test or an unpaired 1-way Analysis of Variance (ANOVA) with Tukey’s Honestly Significant Difference (HSD) post-hoc test where appropriate. A p value ≤ 0.05 was considered significant.

## Abbreviations

ABCC1: ATP-binding cassette protein C1; AKR: Aldo-keto reductase; ANOVA: Analysis of variance; B_max_: Measure of the number of receptors for a particular drug; CBR: Carbonyl reductase; HPLC: High performance liquid chromatography; IC_50_: Concentration required to inhibit growth by 50%; K_app_: Apparent dissociation constant; MIAME: Minimum information associated with a microarray experiment; NQO1: NAD(P)H dehydrogenase, quinine 1; PCR: Polymerase chain reaction; PharmGKB: Pharmacogenomics Knowledgebase.

## Competing interests

The authors declare that they have no competing interests.

## Authors’ contributions

AH participated in writing of the manuscript and performed clonogenic experiments, HPLC experiments, doxorubicin binding assays, and statistical tests. BG performed all microarray experiments and analyzed the microarray data. JS performed the doxorubicin and doxorubicinol localization experiments by confocal microscopy. DM supervised HPLC experiments, including optimization of detection of doxorubicin metabolites. AP devised and supervised the performance of the study, acquired grant funding to support the study, and helped write and revise the manuscript. He is also the corresponding author for this manuscript. All authors read and approved the final manuscript.

## Source of funding

Canadian Institutes of Health Research (Grant MOP-8993 to A.M.P.)

## Pre-publication history

The pre-publication history for this paper can be accessed here:

http://www.biomedcentral.com/1471-2407/12/381/prepub

## Supplementary Material

Additional file 1 **Table S1.**Genes associated with doxorubicin pharmacokinetics or pharmacodynamics in cancer cells or cardiomyocytes as identified in the PharmGKB knowledgebase. Those genes identical to and related to genes significantly changing expression upon acquisition of doxorubicin resistance in MCF-7 breast tumour cells by microarray analysis (false discovery rate of 0.01) are listed in bold regular font and bold italics font, respectively. The fold change in gene expression is also listed for upregulated (+) or down regulated (−) genes. Click here for file

Additional file 2 **Table S2.**Over-representation of doxorubicin pharmacokinetic or pharmacodynamic genes in the dataset of genes associated with the acquisition of doxorubicin resistance in MCF-7 breast tumour cells. The p values assessing the significance of this over-representation are depicted in parentheses, with statistically significant p values listed in bold font. Click here for file

Additional file 3 **Table S3.**Differences in doxorubicin and doxorubicinol DNA binding parameters. DNA binding by doxorubicin or doxorubicinol was compared in an ethidium bromide displacement assay as described in Materials and Methods. The B_max_ and K_app_ values for doxorubicin and doxorubicinol are listed, along with the p values for significant differences in their binding parameters (p < 0.05). Click here for file

Additional file 4 **Table S4.**Primers used for measurement of expression of candidate genes involved in doxorubicin hydroxylation by quantitative PCR. Forward and reverse primers recognizing aldo keto-reductases (AKRs) or carbonyl reductases (CRs) are listed, along with the primers for the reference gene *RPS28*. Click here for file
