# Supplementary material for: Role of aldo-keto reductases and other doxorubicin pharmacokinetic genes in doxorubicin resistance, DNA binding, and subcellular localization
Source: BMC Cancer. 2012 Aug 31;12:381. doi: 10.1186/1471-2407-12-381 (PMC3495881; doi:10.1186/1471-2407-12-381)
Supplement: Additional file 1 — Table S1.Genes associated with doxorubicin pharmacokinetics or pharmacodynamics in cancer cells or cardiomyocytes as identified in the PharmGKB knowledgebase. Those genes identical to and related to genes significantly changing expression upon acquisition of doxorubicin resistance in MCF-7 breast tumour cells by microarray analysis (false discovery rate of 0.01) are listed in bold regular font and bold italics font, respectively. The fold change in gene expression is also listed for upregulated (+) or down regulated (−) genes. [file 1471-2407-12-381-S1.docx]

*Table 1*

| **Gene** | **Forward Primer (5’-3’)** | **Reverse Primer (5’-3’)** |
| --- | --- | --- |
| RPS28 | TCCATCATCCGCAATGTAAAAG | GCTTCTCGCTCTGACTCCAAA |
| AKR1A1 | GTCGGCAGATTGATGACATACTC | TGGCATTCCACCTGCAAGA |
| AKR1B1 | AGCCATGGCAAGCCGTCTC | GCACCACAGCTTGCTGACG |
| AKR1B10 | CAGCCCAGGTTCTGATCCGT | TGGTTGCCATCTCCTCATCA |
| AKR1C RT | N/A | CAGGGCAATCAGGGCTGGGG |
| AKR1C1 | GTAAAGCTTTAGAGGCCAC | ATAAGGTAGAGGTCAACATAA |
| AKR1C2 | GTAAAGCTCTAGAGGCCGT | CTGGTCGATGGGAATTGCT |
| AKR1C3 | GAAGTAAAGCTTTGGAGGTCA | GTCAACATAGTCCAATTGAGC |
| AKR1C4 | CAGAGGTTCCGAGGAACAG | GGACCATCTGTGGTTGAAAGA |
| AKR7A2 | GAGGACAAGGACGGGAAACAGC | CCTGTAGGTCTCAGCCCAGCTA |
| CBR1 | GAAGATTGGCGTCACCGTTC | GTCCCCTTTCCTCTGCTCACT |
| CBR3 | GGAAGGCTGGCCCAACTCAC | GCTTTCCTCTTCTCATCCAGACG |
