# Supplementary material for: Role of aldo-keto reductases and other doxorubicin pharmacokinetic genes in doxorubicin resistance, DNA binding, and subcellular localization
Source: BMC Cancer. 2012 Aug 31;12:381. doi: 10.1186/1471-2407-12-381 (PMC3495881; doi:10.1186/1471-2407-12-381)
Supplement: Additional file 2 — Table S2.Over-representation of doxorubicin pharmacokinetic or pharmacodynamic genes in the dataset of genes associated with the acquisition of doxorubicin resistance in MCF-7 breast tumour cells. The p values assessing the significance of this over-representation are depicted in parentheses, with statistically significant p values listed in bold font. [file 1471-2407-12-381-S2.docx]

*Table 2*

| Cancer Pharmacodynamics | | Cardio Pharmacodynamics | | Pharmacokinetics | | Combined | |
| --- | --- | --- | --- | --- | --- | --- | --- |
| (18) | Fold Change | (24) | Fold Change | (12) | Fold Change | (46) | Fold Change |
| ***ABCA1***  ABCB1  **ABCC1**  ABCC2  ***ABCC13***  ***ABCD3***  **ABCG2**  **CAT**  ERCC2  GPX1  MLH1  MSH2  NFKB1  ***NFKBIZ***  NOS3  **NQO1**  RALBP1  ***SLC22A15***  SLC22A16  SOD1  ***SOD3***  TOP2A  TP53  XDH | 4.39  8.29  -2.62  2.27  2.33  3.58  2.30  3.02  -2.83  -2.47 | **ACO1**  AKR1A1  ***AKR1B1***  ***AKR1B10***  ***AKR1C1***  **AKR1C3**  ATP2A2  ATP5A2  ***ATP2B4***  ATP5B  ATP5C1  ATP5C2  ATP5D  ATP5E  ATP5F1  ATP5H  ATP5I  ***ATP5L***  ***ATP5S***  CBR1  CBR3  CYBA  **CYCS**  NCF4  NOS1  NOS2  NOS3  **RAC2**  RYR2  TOP2B | 2.84  10.03  13.42  4.46  4.71  3.71  2.04  -2.18  2.54  3.68 | AKR1A1  ***AKR1B1***  ***AKR1B10***  ***AKR1C1***  AKR1C2  ***AKR1C3***  CBR1  CBR3  NDUFS2  NDUFS3  NDUFS7  NOS2  NOS3  **NQO1**  POR  XDH | 10.03  13.42  4.46  4.71  3.02 | ***ABCA1***  ABCB1  **ABCC1**  ABCC2  ***ABCC13***  ***ABCD3***  **ABCG2**  **ACO1**  AKR1A1  ***AKR1B1***  ***AKR1B10***  ***AKR1C1***  AKR1C2  **AKR1C3**  ATP2A2  ATP5A2  ***ATP2B4***  ATP5B  ATP5C1  ATP5C2  ATP5D  ATP5E  ATP5F1  ATP5H  ATP5I  ***ATP5L***  ***ATP5S***  **CAT**  CBR1  CBR3  CYBA  **CYCS**  ERCC2  GPX1  MLH1  MSH2  NCF4  NDUFS2  NDUFS3  NDUFS7  NFKB1  ***NFKBIZ***  NOS1  NOS2  NOS3  **NQO1**  POR  **RAC2**  RALBP1  RYR2  ***SLC22A15***  SLC22A16  SOD1  ***SOD3***  TOP2A  TOP2B  TP53  XDH | 4.39  8.29  -2.62  2.27  2.33  2.84  10.03  13.42  4.46  4.71  3.71  2.04  -2.18  3.58  2.54  2.30  3.02  3.68  -2.83  -2.47 |
