# Supplementary material for: Role of aldo-keto reductases and other doxorubicin pharmacokinetic genes in doxorubicin resistance, DNA binding, and subcellular localization
Source: BMC Cancer. 2012 Aug 31;12:381. doi: 10.1186/1471-2407-12-381 (PMC3495881; doi:10.1186/1471-2407-12-381)
Supplement: Additional file 3 — Table S3.Differences in doxorubicin and doxorubicinol DNA binding parameters. DNA binding by doxorubicin or doxorubicinol was compared in an ethidium bromide displacement assay as described in Materials and Methods. The Bmax and Kapp values for doxorubicin and doxorubicinol are listed, along with the p values for significant differences in their binding parameters (p < 0.05). [file 1471-2407-12-381-S3.docx]

*Table 3*

| Pathway | False Discovery Rate = 0.01  Hits (P value) | |
| --- | --- | --- |
|  | Perfect Matches | Perfect or Partial Matches |
| Cancer Pharmacodynamics | 4/18 (0.1573) | 10/18 **(<0.0001)** |
| Cardio Pharmacodynamics | 3/24 (0.0732) | 10/24 **(<0.0001)** |
| Pharmacokinetics | 1/12 (1.0) | 5/12 **(0.0043)** |
| Total | 8/46 **(0.05)** | 20/46 **(<0.0001)** |
