# Supplementary material for: Role of aldo-keto reductases and other doxorubicin pharmacokinetic genes in doxorubicin resistance, DNA binding, and subcellular localization
Source: BMC Cancer. 2012 Aug 31;12:381. doi: 10.1186/1471-2407-12-381 (PMC3495881; doi:10.1186/1471-2407-12-381)
Supplement: Additional file 4 — Table S4.Primers used for measurement of expression of candidate genes involved in doxorubicin hydroxylation by quantitative PCR. Forward and reverse primers recognizing aldo keto-reductases (AKRs) or carbonyl reductases (CRs) are listed, along with the primers for the reference gene RPS28. [file 1471-2407-12-381-S4.docx]

*Table 4*

|  | B_max_ ± SEM | K_app_ (μM) ± SEM |  |
| --- | --- | --- | --- |
| Doxorubicin | | 0.903 ±0.012 | 0.412 ± 0.017 |
| Doxorubicinol | | 0.667 ±0.013 | 0.679 ± 0.034 |
| P value | | <0.0001 | 0.0144 |
